# Editorial Notes

**Published:** 1947

**Authors:** 


### Editorial Notes

**Regional Hospital Board.** THE following will serve on the local Hospital Board, No. 10 Area, South-Western Regional Hospital Area.

CHAIRMAN : Mr. H. G. Tanner (Treasurer, Bristol University).

BRISTOL : Professor R. J. Brocklehurst (Professor of Physiology, Bristol University) ; Mr. E. Cadbury (Treasurer, Bristol Royal Hospital) ; Dr. R. E. Hemphill (Psychiatrist) ; Alderman J. J. Milton (Chairman, Bristol Health Committee) ; Sir Philip Morris (Vice-Chancellor, Bristol University) ; Professor R. M. Walker (Professor of Surgery, Bristol University).

CORNWALL : Mr. C. T. Andrews (Physician, Truro) ; Mr. A. T. C. Bowden (retired Dentist, Liskeard) ; Mrs. M. F. Williams (Chairman, West Cornwall Hospital).

DEVON : Mr. C. P. Brown (Chairman, Prince of Wales Hospital, Plymouth) ; Mr. A. L. Candler (Vice-President, Devon and Exeter Hospital) ; Dr. C. J. Fuller (Physician, Exeter) ; Mrs. K. A. Goddard (Chairman, Exeter Public Health Committee) ; Mr. J. R. Makeig Jones (Chairman, Devon County Nursing Association) ; Mr. J. Marshall (Chairman, Plymouth Health Committee) ; Alderman H. G. Mason (Chairman, Plymouth Reconstruction Committee) ; Brigadier J. Morrison (Chairman, Royal Western Counties Mental Deficiency Institute, Starcross) ; Alderman Mrs. J. M. Phillips (Chairman, Maternity and Child Welfare Committee, Devon County Council) ; Mr. J. W. G. H. Riddell (Gynæcologist, Plymouth).

GLOUCESTERSHIRE : Mr. G. G. Gilmour-White (Chairman, Cheltenham Hospital) ; Mr. W. Niccol (Ophthalmic Surgeon) ; Colonel H. B. Stokes (Gloucestershire County Council).

SOMERSET : Alderman W. Barratt (Chairman, Bath Health Committee) ; Mr. S. McClements (General Practioner, Wellington) ; Mr. S. C. Morland (Chairman, Somerset Public Health Committee) ; Mr. P E. Russell (Chairman, Weston Hospital) ; Miss R. C. Shackles (Matron, Royal United Hospital, Bath).

WILTSHIRE : Miss M. F. Awdry (Vice-Chairman, Visiting Committee, Wilts County Council Mental Hospitals Association).

The Composition of the Board gives general satisfaction. The appointment of Mr. H. G. Tanner as Chairman is particularly welcome. All regions are represented and the members have such experience of Hospital Administration that leaves little room for doubt that they will carry out their work with distinction.

**German Educational Reconstruction**

WE have received an appeal to our readers for books, pamphlets, periodicals and educational equipment for Germany: "Germany has been culturally isolated for the past fourteen years. The need for books and periodicals is desperate, particularly those dealing with educational, technical, political and economic subjects. Many people have a spare book or two on their bookshelves, or periodicals, for which they have no further use." Anyone wishing to help should write to the Secretary, G. E. R., 15 James Street, Long Acre, London, W.C.2.

